# Assessment of the structural and functional characteristics of human mesenchymal stem cells associated with a prolonged exposure of morphine

**DOI:** 10.1038/s41598-021-98682-6

**Published:** 2021-09-28

**Authors:** Francesco Carano, Gabriella Teti, Alessandra Ruggeri, Francesca Chiarini, Arianna Giorgetti, Maria C. Mazzotti, Paolo Fais, Mirella Falconi

**Affiliations:** 1 Department of Biomedical and Neuromotor Sciences, University of Bologna, Via Irnerio 48, 40126 Bologna, Italy; 2 CNR-National Research Council of Italy, Institute of Molecular Genetics “Luigi Luca Cavalli-Sforza”, Unit of Bologna, 40136 Bologna, Italy; 3 IRCCS Istituto Ortopedico Rizzoli, 40136 Bologna, Italy; 4 Department of Medical and Surgical Sciences, University of Bologna, Via Irnerio 49, 40126 Bologna, Italy; 5 Department of Experimental, Diagnostic and Specialty Medicine, University of Bologna, Via Irnerio 48, 40126 Bologna, Italy

**Keywords:** Cell biology, Stem cells

## Abstract

The discovery of the expression of opioid receptors in the skin and their role in orchestrating the process of tissue repair gave rise to questions regarding the potential effects of clinical morphine treatment in wound healing. Although short term treatment was reported to improve tissue regeneration, in vivo chronic administration was associated to an impairment of the physiological healing process and systemic fibrosis. Human mesenchymal stem cells (hMSCs) play a fundamental role in tissue regeneration. In this regard, acute morphine exposition was recently reported to impact negatively on the functional characteristics of hMSCs, but little is currently known about its long-term effects. To determine how a prolonged treatment could impair their functional characteristics, we exposed hMSCs to increasing morphine concentrations respectively for nine and eighteen days, evaluating in particular the fibrogenic potential exerted by the long-term exposition. Our results showed a time dependent cell viability decline, and conditions compatible with a cellular senescent state. Ultrastructural and protein expression analysis were indicative of increased autophagy, suggesting a relation to a detoxification activity. In addition, the enhanced transcription observed for the genes involved in the synthesis and regulation of type I collagen suggested the possibility that a prolonged morphine treatment might exert its fibrotic potential risk, even involving the hMSCs.

## Introduction

Morphine is still widely employed in medical practice to provide postoperative analgesia, as well as to treat patients suffering from severe and chronic pain. The pharmacological effect of morphine is exerted through cell excitability inhibition, mediated by the interaction between the drug and the µ-opioid receptors (MORs) located on the surface of the nervous cells along with the δ and κ (DORs and KORs respectively).

Interestingly, MORs, DORs and KORs were found variedly expressed even in different kinds of human cell populations, such as: keratinocytes, fibroblasts and immune cells^1–3^ and their activation in response to endogenous peptide opioids binding, such as endorphins, dynorphins and enkephalins play a key role in influencing skin homeostasis and tissue repair^4^. Consequently, several studies were then performed to elucidate the impact of morphine in tissues and differentiated cells, as a potential therapeutic option in promoting wound healing.

Although in vivo and in vitro experiments highlighted a positive influence exerted by morphine in improving the tissue healing process by stimulating cell proliferation and angiogenesis^5–7^, the continuous exposition resulted instead in a significant angiogenesis suppression as well as a decreased neutrophil and macrophage recruitment, leading to a decreased release of pro-inflammatory and pro-angiogenic factors and consequently to a delayed tissue regeneration^8–10^. In addition, the prolonged morphine treatment was related to collagen biosynthesis promotion in stellate cells, mesangial cells and myofibroblasts, resulting in the occurrence of fibrotic processes in the liver, kidney and in healing wounds, respectively^11–13^.

Human mesenchymal stem cells (hMSCs) are a versatile class of multipotent adult stem cells capable of self-renewal and osteogenic, chondrogenic, adipogenic as well as myogenic differentiation^14,15^. Because of their trophic property in secreting a wide range of growth factors including: vascular endothelial growth factor, epithelial growth factor, insulin-like growth factor, transforming growth factor-alpha and -beta1 (TGFB1), along with their immunomodulatory, anti-apoptotic and anti-inflammatory properties exerted in response to a mechanical damage or flogosis, they play a fundamental role in tissue repair and regeneration, thus representing an ideal tool in the medical field^16,17^. In this regard, as Holan et al. recently demonstrated, the expression of opioid receptors on hMSCs and how these showed functional characteristics impairment following acute morphine exposition^18^. Nonetheless, little is currently known about the effects on hMSCs resulting from a prolonged use of morphine. Long-term opioid therapy is defined as a daily drug administration for at least ninety days^19^ which is even applied to treat the non-neoplastic and cancer-related pain caused by nociceptive and neuropathic pain conditions, as well as chronic wounds for which topical morphine is a therapeutic strategy. Therefore, we designed the following in vitro study with the aim of assessing the deleterious potential effect on functional characteristics of hMSCs resulting from prolonged morphine treatment, evaluating the cell viability and apoptosis, the proliferative potential, senescence, oxidative stress and autophagic activity in association with the ultrastructural analysis by transmission electron microscopy. In addition, we also focused our attention on the pro-fibrotic influence exerted by morphine, evaluating the expression of the related genes involved in the collagen biosynthesis and its regulation.

## Results

### hMSCs viability after morphine sulphate exposition

**Figure 1:**
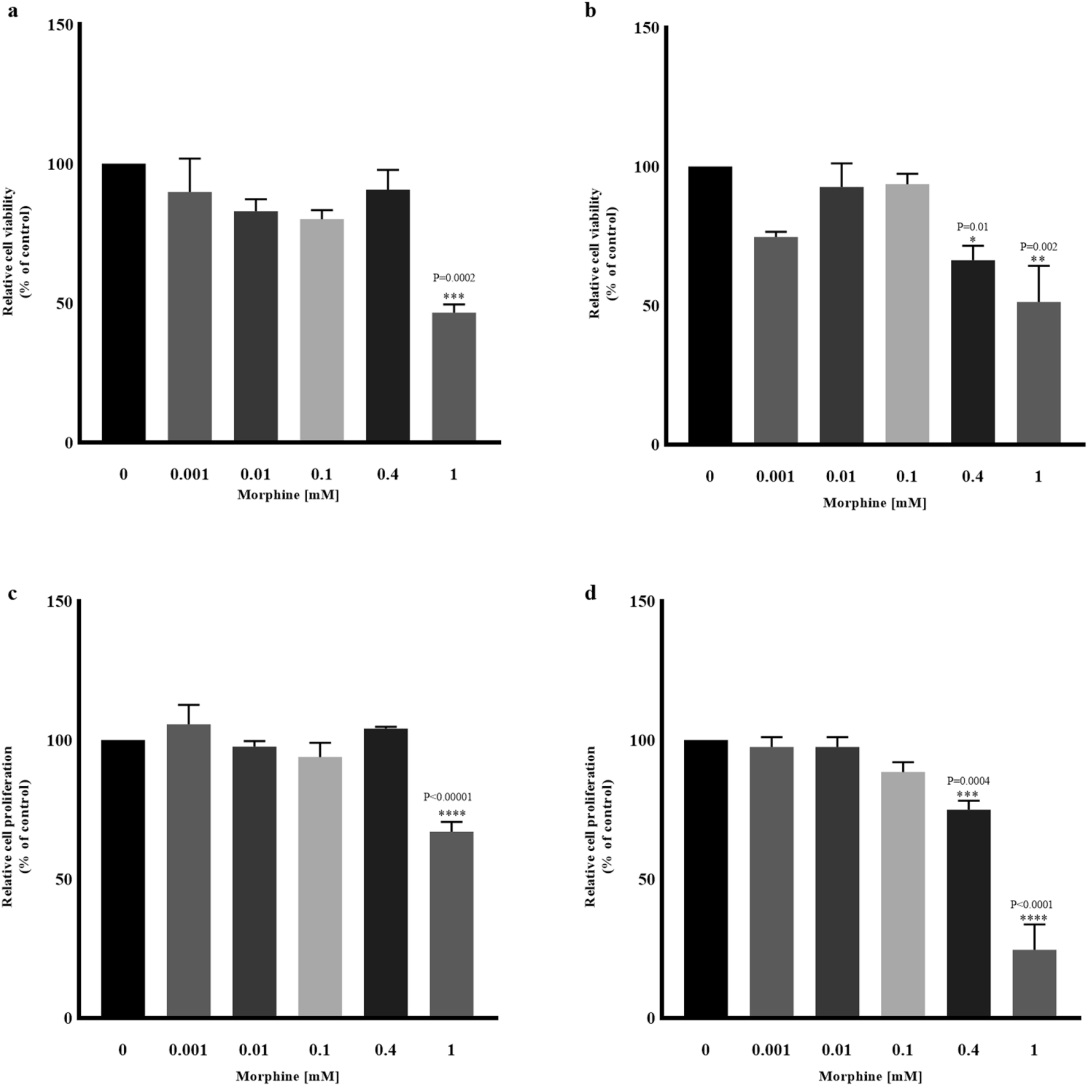
Relative cell viability and proliferation, after nine and eighteen days of exposition in a concentration range from 0.001 mM to 1 mM. The reported values are expressed as mean ± SD of three independent experiments and represented as percentage of the non-treated sample (0 mM). (**a**) Nine days of morphine exposition 1 mM induced a cell viability reduction of 50%. (**b**) Eighteen days induced a 70% and 51% of viable cells population, respectively at 0.4 mM and 1 mM. (**c**) Nine days of 1 mM morphine sulphate induced a 67% proliferation reduction. (**d**) Eighteen days of morphine exposition induced a 74 and 42% proliferation reduction respectively at 0.4 mM and 1 mM.

Nine days of continuous morphine exposure induced a decline of 50% of viability in hMSCs at the concentration of 1 mM (*P* = 0.0002) (Fig. 1a). On the contrary, eighteen days displayed a 70% and a 51% of viable cells, respectively at 0.4 mM (*P* = 0.01) and 1 mM (*P* = 0.002) when compared with the non-treated sample (0 mM) (Fig. 1b).

### Proliferation and cell cycle analysis in hMSCs exposed to morphine sulphate

Compared to the non-treated sample (0 mM), hMSCs exposed to morphine sulphate for nine days showed a significant reduction in cell proliferation only at 1 mM (67% of cell proliferation) (*P* < 0.0001) (Fig. 1c). On the contrary, proliferating cells declined up to 74% and 42% when treated respectively at 0.4 mM (*P* = 0.0004) and 1 mM (*P* < 0.0001) after eighteen days (Fig. 1d).

In order to better investigate in which phase of the cell cycle long term morphine sulphate exposition arrested cells, propidium iodide (PI) staining followed by flow cytometry analysis was carried out.

**Figure 2:**
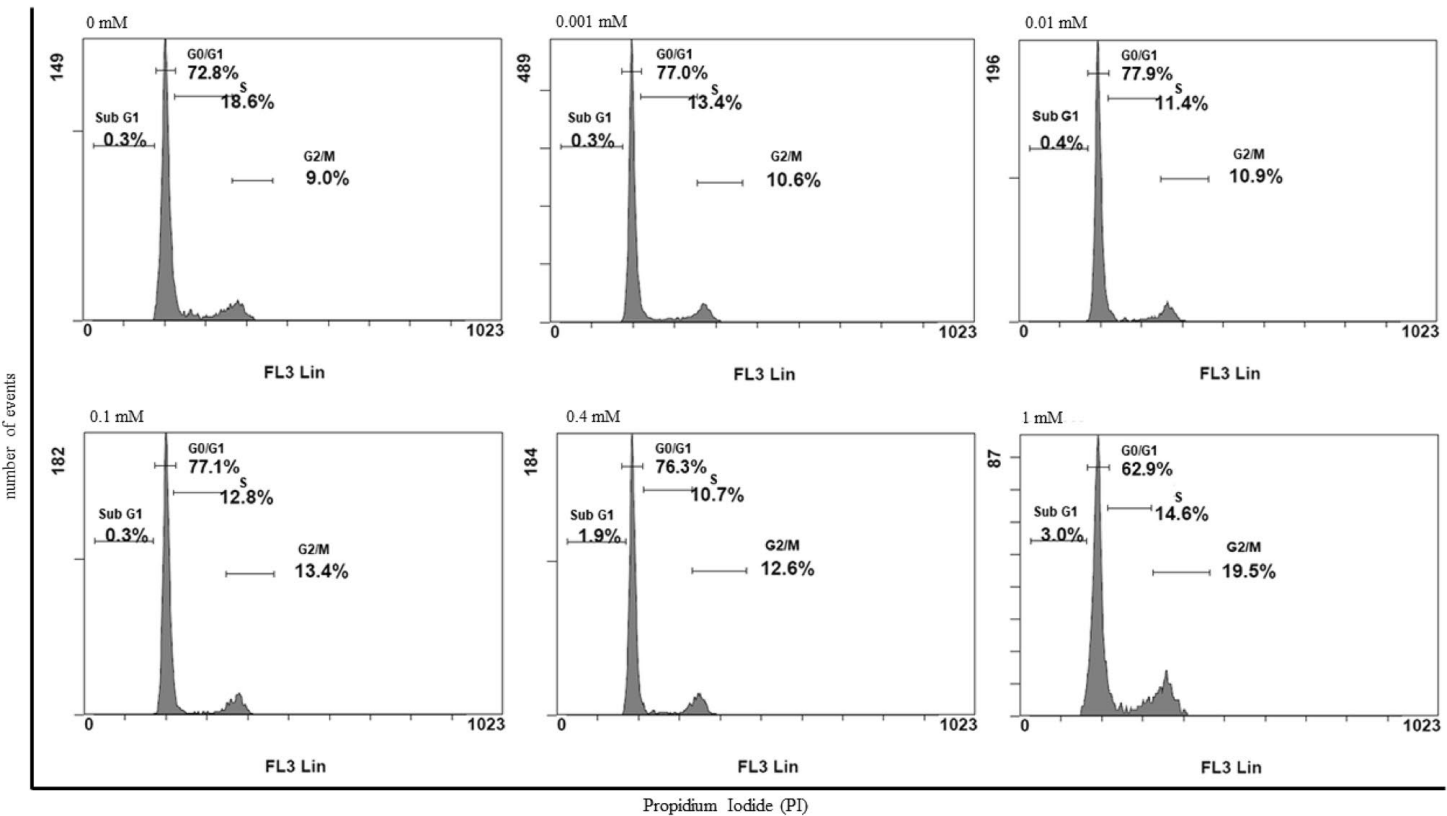
Representative results of cell cycle distribution of hMSCs treated with increasing doses of morphine sulphate for eighteen days and obtained by PI staining for flow cytometry. Graphs show changes in the percentage of cells at G1/0, S and G2/M in relation to the tested concentrations.

The flow cytometry analysis of PI-stained cells showed a moderate decrease of cells in the G0/G1 phase of the cell cycle ranging from 73.3 ± 0.6% (0 mM) to 63.7 ± 1.1% (1 mM) (Fig. 2 and Table 1), while a significant increase of hMSCs were observed in the G2/M phase of the cell cycle, ranging from 8.5 ± 0.7% (0 mM) to 18.8 ± 1.1% (1 mM) (Fig. 2 and Table 1). These data suggested a G2/M arrest of hMSCs induced by morphine sulphate exposition.

**Table 1:** Flow cytometry analysis carried out after eighteen days of 1 mM morphine sulphate exposition highlighted significant differences in the observed cells percentage respectively in G0/G1(^a^*P* < 0.0001) and in G2/M (^b^*P* = 0.0001), suggesting 1 mM of morphine sulphate induces a cell cycle block in this latter phase. The reported values are expressed as mean ± SD of three independent experiments.

Morphine sulphate	G_0_/G_1_ (%)	S Phase (%)	G_2_/M (%)
0 mM	73.3 ± 0.6	18.7 ± 0.1	8.5 ± 0.7
0.001 mM	76.8 ± 0.3	13.6 ± 0.2	10.2 ± 0.6
0.01 mM	77.9 ± 0.1	11.9 ± 0.7	10.3 ± 0.8
0.1 mM	77.5 ± 0.5	12.4 ± 0.6	12.8 ± 0.9
0.4 mM	76.7 ± 0.6	10.8 ± 0.1	11.9 ± 1.1
1 mM	63.7 ± 1.1^a^	15.3 ± 1.0	18.8 ± 1.1^b^

### Senescence-associated beta-galactosidase (SA-β-gal) activity in hMSCs exposed to morphine sulphate

**Figure 3:**
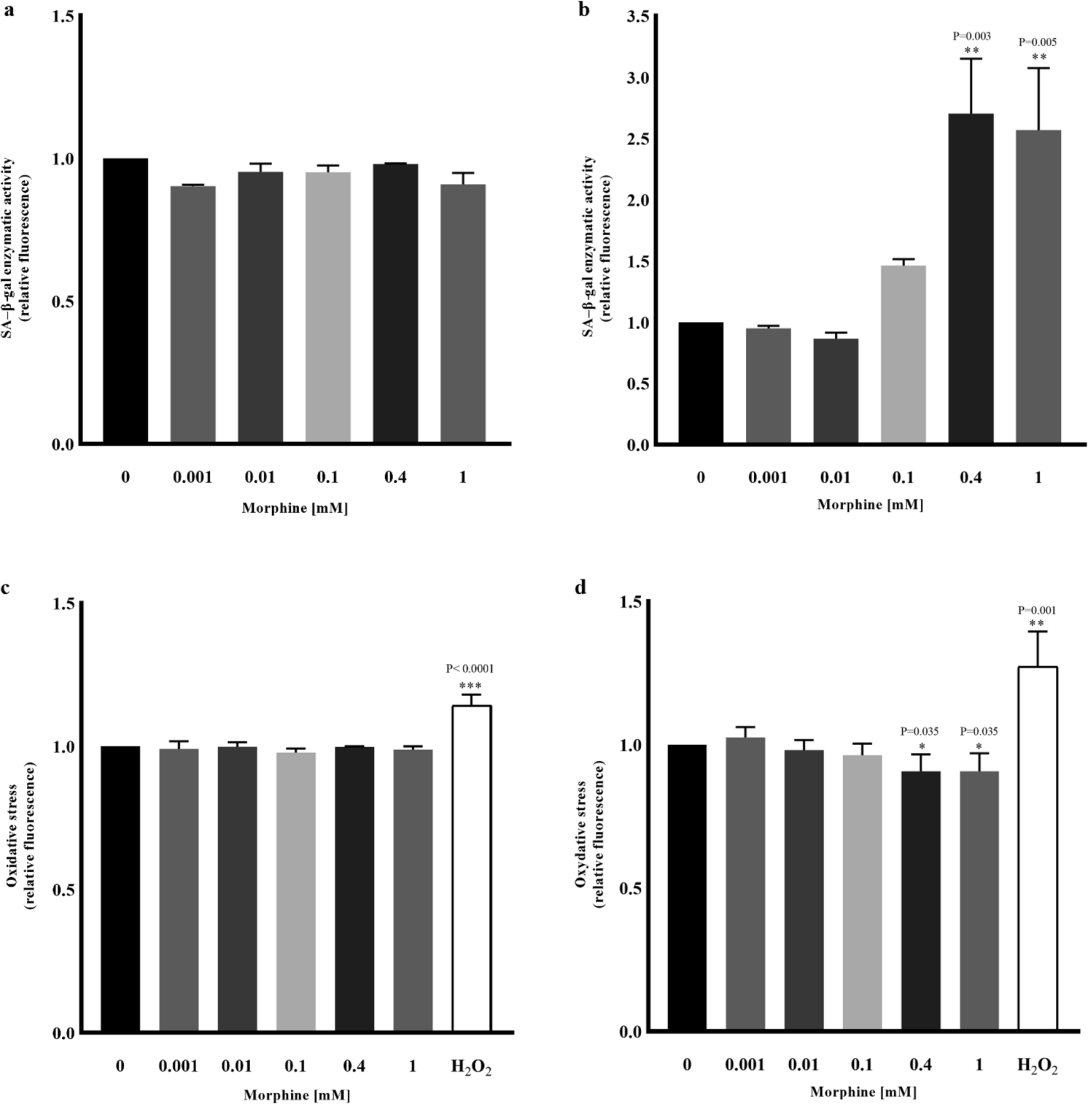
SA-β-Gal assay and ROS analysis carried out after nine and eighteen days of morphine exposition. The reported values are expressed as mean ± SD of three independent experiments and represented as fluorescence variations compared to non-treated sample (0 mM). (**a**) Nine days of morphine treatment did not induce any increase of SA-β-Gal enzymatic activity. (**b**) Eighteen days of morphine treatment induced on the contrary, a marked enzymatic activity. (**c**) Nine days of morphine exposition did not induce significant oxidative stress effect. (**d**) Compared to the non-treated sample, eighteen days displayed oxidative stress decrease both at 0.4 and 1 mM. White bar represented in both plot refers to hydrogen peroxide used as positive control.

No significant variation in SA-β-Gal activity was observed after nine days of drug exposition (Fig. 3a), compared to the non-treated sample (0 mM). On the contrary, 0.4 mM and 1 mM (*P* = 0.003 and *P* = 0.005) induced a marked enzymatic activity after eighteen days of morphine sulphate exposition (Fig. 3b).

### Reactive Oxygen Species (ROS) analysis in hMSCs exposed to morphine sulphate

hMSCs exposed to different concentrations of morphine sulphate showed no significant differences in ROS detection after nine days of treatment (Fig. 3c), while a slight but significant reduction of ROS is detected in hMSCs treated with 0.4 mM and 1 mM (*P* = 0.035) compared to the non-treated sample (0 mM) (Fig. 3d).

### Ultrastructural analysis by TEM

**Figure 4:**
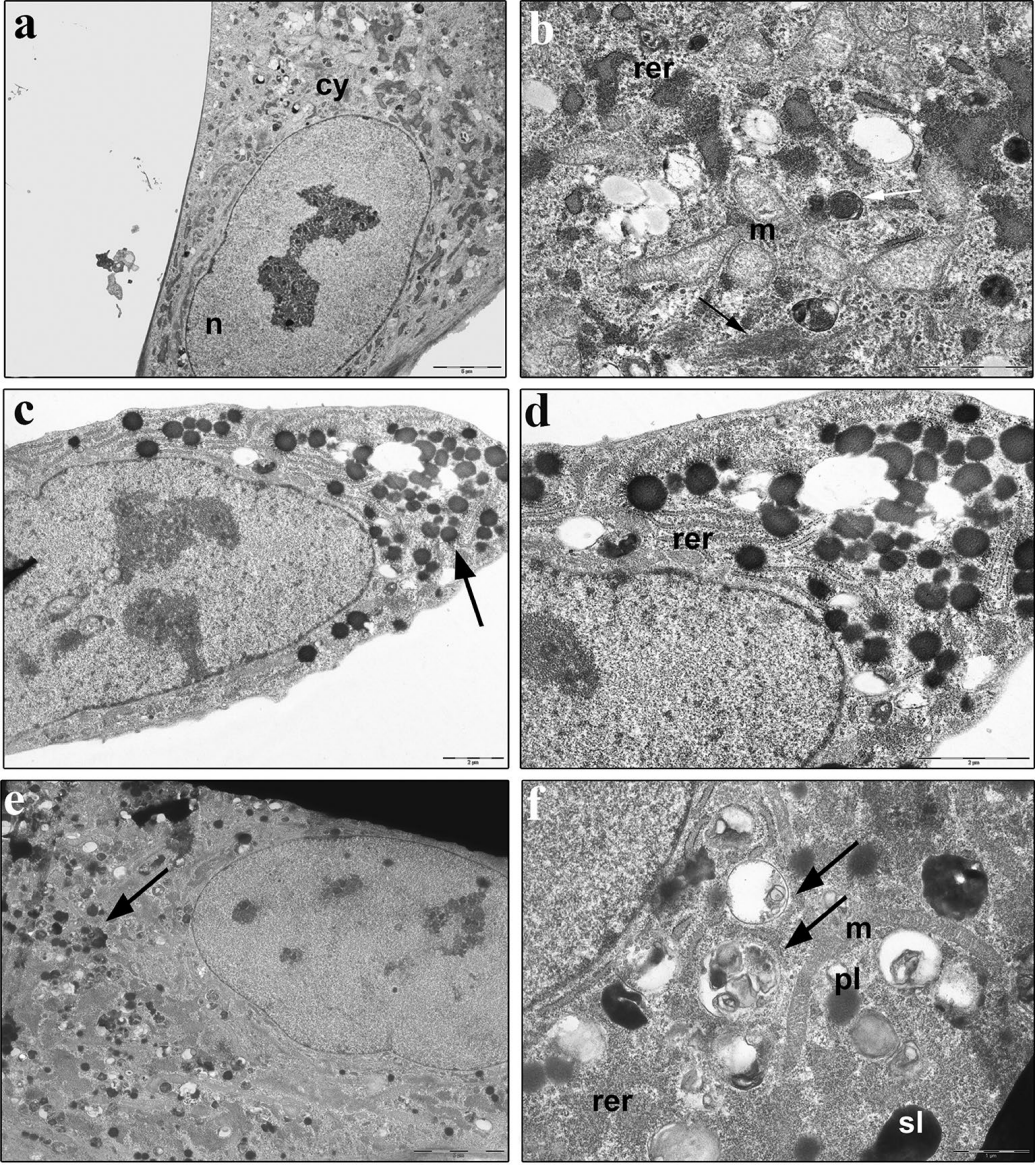
TEM ultrastructural analysis of hMSCs treated with morphine sulphate for eighteen days. (**a**) non-treated hMSCs show a polygonal morphology, with nucleus (n) and cytoplasm (cy) well preserved (bar: 5 µm). (**b**) Detail of cytoplasm showing long shape mitochondria (m), rough endoplasmic reticulum (rer), Golgi complex (black arrow) and primary lysosomes (white arrow) (bar: 2 µm); (**c**) hMSCs exposed to 0.4 mM of morphine sulphate. Several lysosomes (black arrow) are observed in the cytoplasm (bar: 2 µm); (**d**) detail of cytoplasm showing lysosomes, some vesicles and well developed RER (rer) (bar: 2 µm); (**e**) low magnification image of hMSCs exposed to 1 mM of morphine sulphate. Several lysosomes (black arrow), and vesicles are still observed in the cytoplasm (bar: 5 µm; (**f**) detail of cytoplasm showing several primary (pl) and secondary lysosomes (sl), autophagic vesicles (black arrows) and enlarged RER (rer) are detected. Long shape and well-preserved mitochondria (m) are easily observed (bar: 1 µm).

A low magnification image of non-treated (0 mM) hMSCs showed a preserved morphology, with nucleus and nucleoli well detected (Fig. 4a). Several mitochondria, numerous rough endoplasmic reticulum, Golgi complex and a few lysosomes were detected in the cytoplasm, indicating cells characterized by high protein synthesis and secretive activity (Fig. 4b).

hMSCs treated with 0.4 mM of morphine sulphate retained their polygonal morphology and a well preserved nucleus and cytoplasm (Fig. 4c). At low magnification several lysosomes were detected in the cytoplasm (Fig. 4c), while RER and mitochondria showed no changes in their morphology and organization (Fig. 4d). Cells exposed to 1 mM of morphine sulphate showed a polygonal morphology, with the cytoplasm still filled by several lysosomes (Fig. 4e). At higher magnification, enlarged and dilated RER, several autophagic vesicles, primary and secondary lysosomes were detected (Fig. 4f). Long shape mitochondria with preserved internal cristae were observed (Fig. 4f).

### Autophagy and apoptosis-related protein expression

In order to confirm the activation of autophagy, as demonstrated by TEM analysis after eighteen days of morphine sulphate exposition, western blot assay was carried out to investigate the expression of autophagy-related markers beclin-1 (BECN1) and microtubule-associated proteins 1A/1B light chain 3B (LC3) in cells exposed to the same treatment duration.

**Figure 5:**
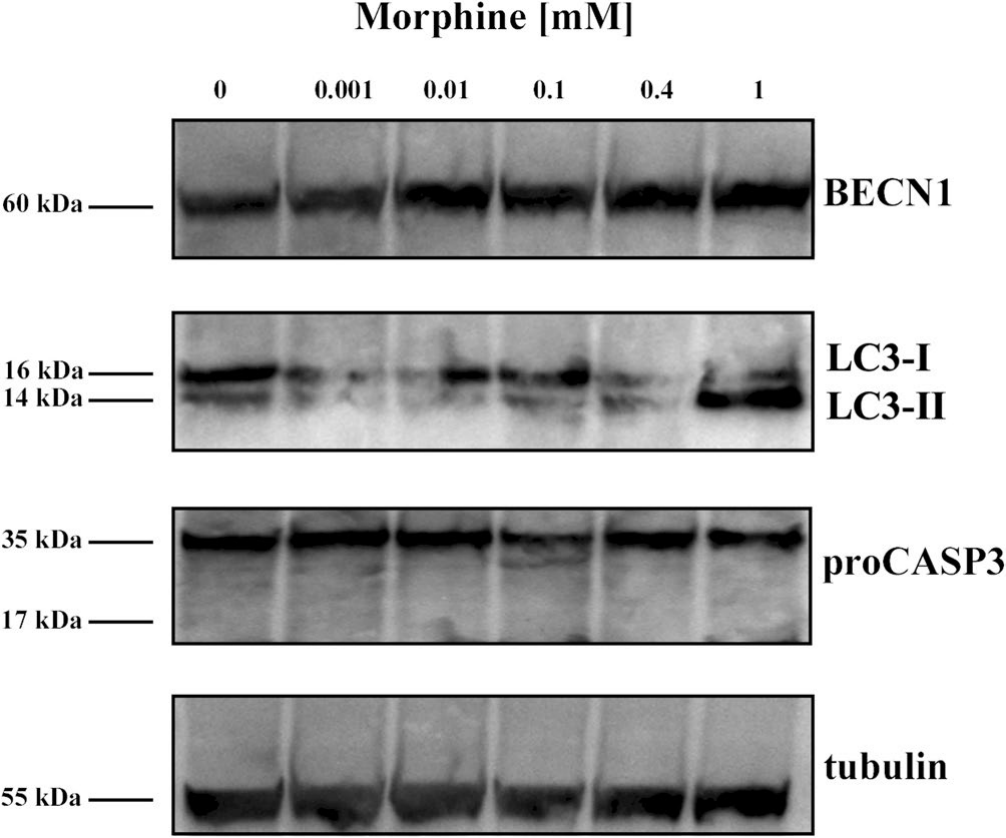
Cropped images representative of western blot analysis showing the expression of autophagic related markers BECN1, LC3 (LC3-I and LC3-II) and the apoptotic related marker proCASP3/CASP3, in hMSCs exposed to different concentration of morphine sulphate for eighteen days. Original blots representative of each tested marker are included in Supplementary Figure S1. To hybridize each marker separately, blots were cut based on the molecular weight of the proteins and empty lanes were removed by trimming the edges.

**Figure 6:**
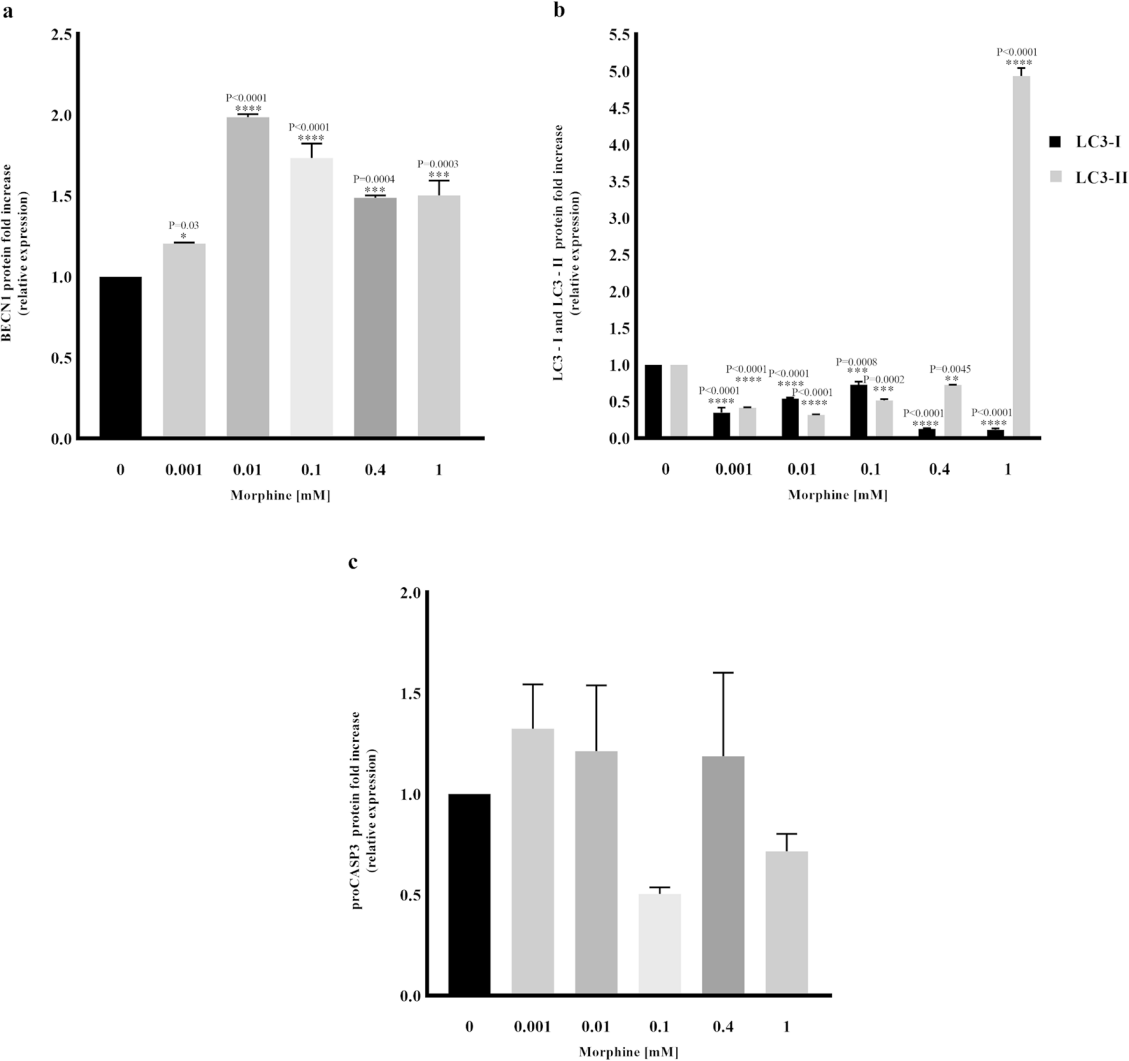
Densitometric analysis of western blot data, carried out after eighteen days of morphine sulphate exposition. The reported values are expressed as mean ± SD of three independent experiments and represented as fold changes compared to non-treated samples (0 mM). Relative amounts of BECN1, LC3 (LC3-I and LC3-II) and proCASP3/CASP3 protein expression were normalized to the intensity of tubulin protein. (**a**) Morphine sulphate induced a BECN1 expression enhancement already at 0.001 mM in a dose–response manner. (**b**) 1 mM morphine exposition caused a five-fold LC3-II fragment upregulation (**b**). Both results suggest that prolonged exposure of morphine may induce an increase of autophagy. (**c**) The presence of unvaried levels of proCASP3 suggests low or absent levels of apoptosis.

Results showed a dose-dependent BECN1 increased expression already from 0.001 mM concentration (*P* = 0.03), up to 1.5 increase at 1 mM (*P* = 0.0003) compared to non-treated (0 mM) samples (Figs. 5, 6a). The analysis of the LC3 marker confirmed an upregulation of autophagy, demonstrated by a strong signal of the LC3-II protein fragment in cells exposed to 1 mM of morphine (Fig. 5). Quantitative analysis confirmed a five-fold increase of LC3-II fragment compared to non-treated samples (Fig. 6b).

In order to investigate the induction of cell death by apoptosis, the expression of procaspase-3 (proCASP3) and the cleaved fragment caspase-3 (CASP3) was investigated. Figure 5 showed the same levels of proCASP3 in all the samples, while no cleaved fragment corresponding to CASP3 activation was observed (Figs. 5, 6c), suggesting the lack or a low undetectable level of apoptosis as mechanism of cell death.

### m-RNA expression analysis by qRT-PCR in hMSCs exposed to morphine sulphate

**Figure 7:**
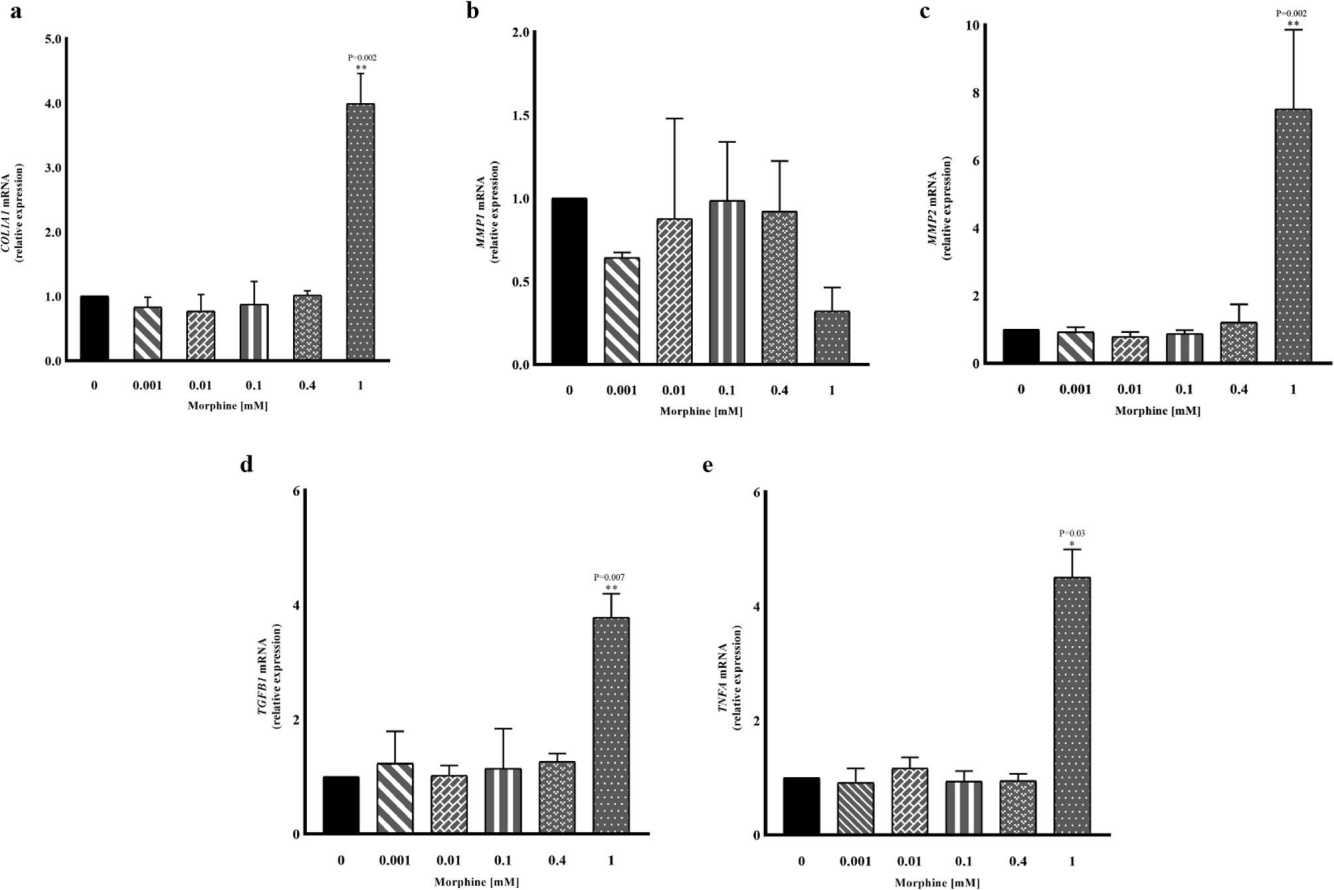
qRT – PCR evaluating the effect of different concentrations of morphine sulphate on *COL1A1*, *MMP1*, *MMP2*, *TGFB1* and *TNFA* gene expression, in human hMSCs exposed for eighteen days. The reported values are expressed as mean ± SD of three independent experiments and represented as fold changes compared to non-treated samples (0 mM). (**a**) 1 mM Morphine sulphate induces a fourfold increase of *COL1A1* mRNA levels. (**b**) 1 mM morphine treatment induced a 0.5 fold downregulation although not statistically significant of mRNA *MMP1* mRNA in hMSCs, and a sevenfold *MMP2* gene upregulation (**c**). Eighteen days of 1 mM morphine sulphate exposition displayed fourfold expression increase of both *TGFB1* (**d**) and *TNFA* (**e**).

Eighteen days of treatment with morphine sulphate did not show any significant variations in collagen type I alpha 1 (*COL1A1*) mRNA expression in hMSCs up to 0.4 mM. On the contrary, a fourfold increase of mRNA *COL1A1* was observed at 1 mM morphine sulphate (*P* = 0.002) (Fig. 7a). Long term exposition did not significantly alter the amount of matrix metalloproteinase-1 (*MMP1*) mRNA (Fig. 7b) although 1 mM displayed a visible mRNA decrease. Conversely, 1 mM morphine sulphate induced a significant sevenfold upregulation of matrix metalloproteinase-2 (*MMP2*) mRNA expression (*P* = 0.002) (Fig. 7c) compared to non-treated samples (0 mM).

1 mM of morphine sulphate incubated with hMSCs for eighteen days induced a threefold upregulation of the *TGFB1* mRNA expression (*P* = 0.007) (Fig. 7d) and a fourfold increase of the tumour necrosis factor-alpha (*TNFA*) mRNA expression (*P* = 0.03) (Fig. 7e).

## Discussion

The characterization of opioid receptors in myofibroblasts and keratinocytes raised the question of how the clinical morphine administration could impact the tissue regeneration. Although the therapeutic concentrations were found to improve the wound healing process by stimulating angiogenesis and promoting cell proliferation, prolonged use instead resulted in the risk of systemic fibrosis as well as delay in wound healing mainly caused by angiogenesis suppression along with a prolonged inflammatory phase associated with oxidative stress. hMSCs play a fundamental role in the tissue repair process due to their ability to migrate into injured sites, differentiating in a wide variety of cell types and secreting immunoregulatory molecules and growth factor, thus representing an ideal tool in regenerative medicine. Opioid receptors were recently characterized even in hMSCs—in this regard the acute morphine exposition induced a proliferation decrease in a dose response fashion without affecting the metabolic activity^18^. In our work, we evaluated the effects of a long-term treatment carried out after nine and eighteen days, respectively. The duration of the morphine exposition was chosen with the aim of overcoming the intrinsic ability of hMSCs to maintain their functional integrity^20^. Although we did not find an evident relation between long-term morphine exposition and apoptosis, we observed a marked cell viability decrease as well as a cytostatic effect for the highest concentrations tested. In order to elucidate the mechanism behind these detrimental effects, we assumed that a prolonged treatment could generate biosynthesis of ROS, which can lead over time to single and double strand DNA damage and telomere attrition. It is well-known that these damaging stimuli induce cellular senescence, characterized by an irreversible state of cell cycle arrest as well as a permanent loss of replicative capacity^21,22^. In this regard, senescent hMSCs display functional decline and a reduced migratory capacity, thus resulting in tissue regeneration impairment^23^. Since in vivo studies carried out in murine models have reported a significant oxidative stress increase following morphine administration^24^, we hypothesized that long-term exposition might induce hMSCs senescent phenotype through ROS accumulation.

SA-β-Gal assay, tested on hMSCs exposed for eighteen days, showed an increase in enzyme activity, suggesting a senescent state in cells exposed to 0.4 mM and 1 mM of morphine sulphate. This result is in agreement with the proliferation and flow cytometry analysis which demonstrated respectively a reduction of cell proliferation and the presence of a G2/M arrest of the cell cycle induced by highest concentrations of morphine treatment.

Contrary to our expectations, ROS levels were reduced in cells long exposed to 1 mM morphine sulphate. This contradictory result could be explained by the increased protein expression of BECN1 and LC3-II fragment, as well as by the presence of several lysosomes and autophagic vesicles in hMSCs exposed long-term to morphine sulphate, demonstrated by the ultrastructural analysis.

Autophagy is an intracellular lysosomal degradation process by which dysfunctional components are removed by the cell. Autophagy is a major sensor of redox signalling. ROS function as intracellular messengers at physiologically low level, whereas excessive production of ROS causes oxidative damage to cellular constituents and thus induces autophagy to maintain cellular homeostasis^25^. In our system, morphine exposition induced ROS production which is balanced by an upregulation of the autophagic process.

A prolonged morphine exposure is related to enhanced collagen biosynthesis in stellate cells, mesangial cells and myofibroblasts, thus resulting in a risk of fibrotic processes in the liver, kidneys and wound healing progression, respectively^11^^;^^26,27^. As the hMSCs are able to self-produce the collagen^28^, we investigated if a continuous morphine exposition might lead to an impaired biosynthesis.

Since the observed effects were more pronounced for more than nine days, we decided to assess eventual collagen expression impairment only at the longest period of exposition included in our study. In this regard, we quantified the *COL1A1* transcript as well as the related positive regulatory gene *TGFB1*^29^ and in particular *MMP1* and *MMP2* mRNA, whose mRNA variation is considered a valid biomarker of fibrosis for its role in degrading collagen^30^.

The significant increased *COL1A1* and *TGFB1* transcript levels observed at the highest morphine concentration tested led us to speculate that a prolonged drug treatment enhances the collagen expression even in hMSCs, through *TGFB1* upregulation.

Our findings are consistent with previous in vivo studies, where mice subjected to chronic administration showed a significant correlation between the length of drug treatment and the extracellular matrix deposition^12^. In addition, the transforming growth factor-beta family modulates different aspects of cell regulation and survival, including cell proliferation inhibition and cellular senescence^31,32^.

Since the literature suggests an inverse correlation between the MMP1 and systemic sclerosis^33,34^, we hypothesized a possible decrease in *MMP1* mRNA levels, especially in relation to the inhibitory effect on this gene exerted by the *TGFB1* expression^35^. Although the transcript levels observed within the group treated with 1 mM of morphine after eighteen days might suggest a *MMP1* gene inhibition, the statistical comparison with the non-treated cells did not display statistically significant differences. On the contrary, 1 mM morphine showed a significant sevenfold *MMP2* mRNA increase, whose trend is coherent with the direct correlation observed during the progression of hepatic and cardiac fibrosis^36^. Finally, we decided to evaluate the pro-inflammatory cytokine *TNFA* transcription level, as a component of the senescent associated secretory phenotype (SASP). SASP consists in the secretion of pro-inflammatory signals in the tissue microenvironment and contributes to age-related disorders^37^. Indeed, senescent cells prompt their fibrogenic actions primarily by secreting SASP components. Kidney fibrosis^38^ and idiopathic pulmonary fibrosis^39^ have been associated to the presence of senescent cells and their SASP factors. As expected, we detected high levels of *TNFA* mRNA in hMSCs exposed to morphine sulphate, in agreement with a senescent phenotype, which could have a key role in stimulating a fibrogenic action.

In conclusion, the prolonged exposure of morphine may result to a marked cell viability decrease, and cellular senescence, as demonstrated from the observed G2/M phase arrest in combination with the increased levels of SA-β-Gal.

The *COL1A1* upregulation suggests a collagen biosynthesis enhancement mediated by a direct stimulation of the *TGFB1* gene. Although this result could be indicative of an increased extracellular matrix deposition which could lead to systemic fibrosis risk, as supported by the high *MMP2* mRNA levels, the observed alterations occurred only at the highest concentration tested. Consequently, we assume that long-term morphine therapy, especially in relation with high dosage to treat the nociceptive and neuropathic pain, as well as those deriving from chronic wounds, may negatively impact the hMSCs regenerative potential and the correct outcome of wound healing. In particular, the induced upregulation of *TNFA* might lead to a prolonged inflammatory phase, which may eventually result in a delayed tissue repairing process.

## Materials and methods

### hMSCs isolation and culture condition

hMSCs were isolated from the umbilical cords of full-term pregnancies, donated by the “Cord blood tissue bank and biobank”, Sant’Orsola – Malpighi Hospital, Bologna (Italy). Informed consent was obtained from each patient according to the guidelines of the National Bioethics Committee, and the samples were collected following a protocol approved by the Ethics Committee of the Sant’Orsola- Malpighi Hospital.

Wharton’s jelly samples were cut into small fragments, washed in PBS several times and cultured in Minimal Essential Medium (MEM) (Gibco, Thermo Fisher Scientific Monza, Italy) supplemented with 10% FBS, 1% penicillin and streptomycin (Gibco, Thermo Fisher Scientific) at 37 °C in saturated humidity containing 95% air and 5% CO_2_. The medium was completely replaced every 3 days until the adherent cell population reached about 80% confluence.

The hMSCs utilized for all the experiments have been previously characterized^40^ for the expression of the positive mesenchymal markers CD105, CD73, CD90, and negative hemopoietic markers CD34, CD19, CD45, HLADR using flow cytometric analysis in agreement with minimal criteria for the identification of human hMSCs^41^.

### Morphine sulphate exposition

Once the third passage had been reached, the cells were harvested at log phase and a density of 1 × 10^5^ cells/ml were plated in new 25 cm^2^ tissue-culture flasks containing a final volume of 5 ml MEM supplemented with 10% FBS, 1% Penicillin–Streptomycin (Gibco, Thermo Fisher Scientific) and the designed morphine sulphate dilutions range (0 mM; 0.001 mM; 0.01 mM; 0.1 mM; 0.4 mM and 1 mM). Cells were finally incubated at 37 °C in and 5% of CO_2_. To simulate a prolonged exposition, we tested two treatment groups of nine and eighteen days of morphine administration, respectively, replacing the medium supplemented with the drug every 3 days.

### Cell viability analysis

hMSCs, previously treated with morphine sulphate, were seeded in a 96 well plate at the density of 1 × 10^4^ cells/well in the same experimental conditions as previously described. At the end of the treatment, the medium was changed with a new one containing 0.5 mg/mL of b3-(4,5-dimethyiazol-2-1)-2-5-diphenyl tetrazolium bromide (Sigma-Aldrich, St. Louis, MO, USA), for 3 h at 37 °C in 5% CO_2_. The medium was then discarded and the resulting formazan crystals were dissolved by adding dimethylsulfoxyde in isopropanol (1:1). Colorimetric reaction was finally quantified by microplate reader (LT-4000 Microplate reader, Labtech L.t.d, Heatfield, UK), measuring the absorbance at 578 nm and a reference wavelength of 690 nm.

### Cell proliferation analysis

Proliferation analysis was carried out using Cell Proliferation ELISA Bromodeoxyuridine (BrdU) (Roche Diagnostics GmbH, Mannheim, DE) according to the manufacturer’s protocol. In brief, hMSCs previously exposed to morphine sulphate, were seeded in a 96 well plate at the density of 1 × 10^4^ cells/well and cultured in MEM supplemented with 10% FBS and 10 µM BrdU labelling solution for 24 h at 37 °C in 5% CO_2_. Then, cells were fixed for 30 min and incubated with anti BrdU-peroxidase conjugate antibody at room temperature for 90 min. After three washing steps in PBS, a tetramethyl-benzidine substrate solution was added for 10 min at room temperature (RT), and the reaction was stopped with 1 M H_2_SO_4_. The optical density was measured using a spectrophotometer microplate reader (LT-4000 Microplate reader) at a wavelength of 450 nm and a reference wavelength of 690 nm.

### Cell cycle analysis

Cell cycle analysis was assessed by flow cytometry in combination with PI/RNase, a staining standard procedure. After eighteen days of morphine exposition, 1 × 10^6^ cells were collected for each experimental point, washed with PBS and then resuspended in ethanol 70% and stored at − 20 °C over night. Then, cell suspension was centrifuged at 100 g for 10 min, washed in PBS and resuspended in a solution of PI for at least 30 min. The analysis was carried out by FC500 flow cytometer (Beckman Coulter, Indianapolis, IN, USA) and the results were examined by the appropriate software (version 2.2, CXP, Beckman Coulter). At least 15,000 events per sample were acquired.

### SA-β-Gal enzymatic activity

SA–β‐gal assay was carried out according to the manufacturer's protocol (Thermo Fisher Scientific, Eugene, OR, USA). Briefly, hMSCs previously treated with morphine sulphate were seeded in a 96 well plate at the density of 1 × 10^4^ cells/well in the same experimental conditions as previously described. After 24 h, samples were then fixed with 2% formaldehyde in PBS for 10 min and then stained with the cell event senescent green probe, (Thermo Fisher Scientific) a sensitive fluorescent substrate for the β-galactosidase enzyme for 1 h and 30 min at 37 °C in a free CO_2_ environment. Samples were finally washed in PBS and the fluorescent signal was measured using a fluorescent microplate Reader (GloMax Discover System, GM3000, Promega Corporation, Madison, WI, USA) at an excitation wavelength of 490 nm and an emission wavelength of 514 nm.

### ROS detection assay

hMSCs previously treated with morphine sulphate, were seeded in a 96 well plate at the density of 1 × 10^4^ cells/well in the same experimental conditions as previously described. After 24 h, cell medium was changed with a new one containing 5 µM of 6-carboxy-2′,7′-dichlorodihydrofluorescein diacetate fluorescent dye (Invitrogen, Carlsbad, CA, USA). Positive controls consisted in hMSCs previously treated with 200 µM H_2_O_2_ for 1 h.

Plates were incubated at 37 °C in 5% CO_2_ for 15 min. Lastly, fluorescent dye was removed and cells were replenished with new pre-warmed culture medium FBS free. Intracellular ROS accumulation induced by morphine exposition was quantified by GLOMAX DISCOVER microplate reader (GM3000, Promega Corporation) with excitation and emission wavelengths set respectively at 520 nm and 500/550 nm.

### TEM ultrastructural analysis

hMSCs previously treated with morphine sulphate for eighteen days, were seeded on cover glasses at the density of 3 × 10^4^ cells/well for 24 h following the same cell culture conditions as previously described. Then, the samples were fixed with 2.5% glutaraldehyde in 0.1 M of cacodylate buffer for 2 h at 4 °C and post fixed with a solution of 1% OsO_4_ in 0.1 M cacodylate buffer, for 30 min at RT. Cells were washed with 0.15 M cacodylate buffer, dehydrated in a graded series of acetone and embedded in Epon resin (Sigma Aldrich).

Ultrathin sections were stained with uranyl acetate and lead citrate and observed by transmission electron microscope (FEI Company, Hillsboro, Oregon, USA) at an accelerating voltage of 80 kV. Images were recorded by Megaview III digital camera (FEI Company).

### Western blot analysis

Cellular pellets of were extracted by using RIPA lysis buffer (Thermo Fisher Scientific) supplemented with 25 μmol/L protease inhibitor cocktail (Thermo Fisher Scientific) and 1 μL of β-mercapto-ethanol (Sigma-Aldrich). Total proteins were resolved on 4–12% SDS polyacrylamide gel electrophoresis (SDS–PAGE) and electrophoretically transferred onto a nitrocellulose membrane using a wet blotting apparatus (Thermo Fisher Scientific). The membranes were blocked with 5% bovine serum albumin in TBS—Tween buffer (blocking reagent) (Thermo Fisher Scientific) for 30 min at room temperature and were then incubated with the following primary antibodies: rabbit anti-human Beclin (Cell Signaling Technologies, Euroclone, Milan, Italy); rabbit anti-human LC3 (Cell Signaling Technologies, Euroclone); rabbit anti-human caspase 3 antibody (Cell Signaling Technologies, Euroclone); mouse anti-human tubulin antibody (Sigma- Aldrich).

All the primary antibodies were diluted 1:1000 in blocking reagent and the incubations were performed at 4 °C over night. After washing with TBS-Tween buffer, each blot was incubated with anti-rabbit secondary antibody (1:2000 dilution; Cell Signaling Technology, Euroclone) or anti-mouse antibody (1:2000 dilution; Sigma Aldrich) for 1 h and 30 min at room temperature. The antibody signal was visualized with the enhancement chemiluminescence system (Thermo Fisher Scientific). Images were obtained by using IBRIGHT Western Blot Imaging System (Thermo Fisher Scientific). Band densitometry was determined using ImageJ software (National Institutes of Health), and the intensities of the specific protein bands were corrected for equal tubulin loading.

### Gene expression

hMSCs previously exposed to morphine sulphate were collected and the total RNA was isolated by PURELINK RNA Micro Scale Kit (Thermo Fisher Scientific) and quantified by Nanodrop 2000c Spectrophotometer (Thermo Fisher Scientific). First strand cDNA was synthetized by reverse transcription using SUPERSCRIPT III First-Strand Synthesis System (Thermo Fisher Scientific). The expression of mRNA was analyzed by quantitative Real Time PCR using 7500 Real Time PCR (Thermo Fisher Scientific). For the analysis, the following TAQMAN assays (Thermo Fisher Scientific) were used: *COL1A1* (Hs00164004), *TGFB1* (Hs00998133), *TNFA* (Hs01113624), *MMP1* (Hs00899658) and *MMP2* (Hs01548727).

The relative gene expressions were normalized to glyceraldehyde 3-phosphate dehydrogenase (*GAPDH*; Hs02786624), and the data were presented as the fold change using the formula 2^−ΔΔCT^ as recommended by the manufacturer (User Bulletin No.2 P/N 4,303,859, Applied Biosystems).

### Statistical analysis

Each experiment included in the study was independently repeated three times and single morphine concentrations were tested in triplicate. One-way analysis of variance (ANOVA) followed by Dunnett's multiple comparisons test, were carried out using GraphPad Prism 8.0.1. (GraphPad Software Inc., San Diego, CA, USA). The differences were considered significant at *P* < 0.05.

## Supplementary Information

Supplementary Information.
