# Supplementary material for: Assessment of the structural and functional characteristics of human mesenchymal stem cells associated with a prolonged exposure of morphine
Source: Sci Rep. 2021 Sep 28;11:19248. doi: 10.1038/s41598-021-98682-6 (PMC8478991; doi:10.1038/s41598-021-98682-6)
Supplement: Supplementary file 1 — Supplementary Information. [file 41598_2021_98682_MOESM1_ESM.pdf]

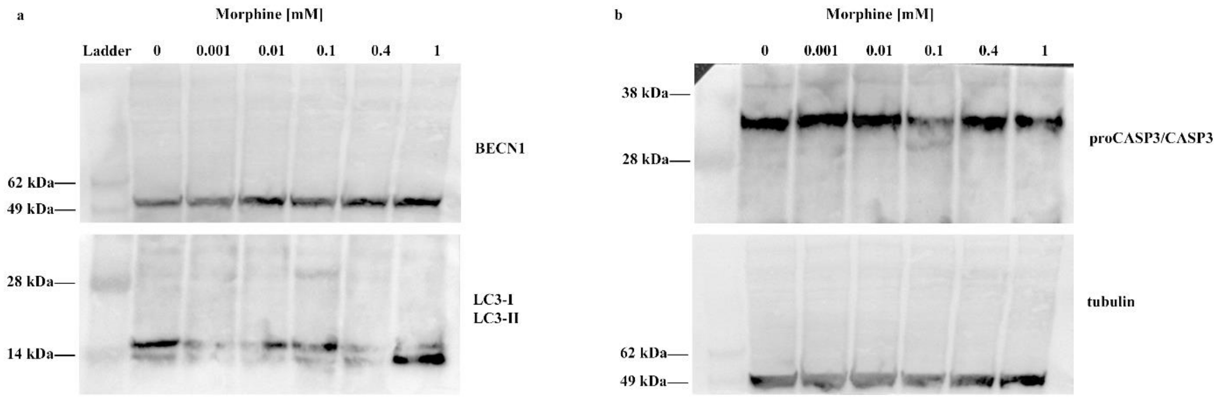

Figure S1. Representative images of full length western blot analysis of (**a**) autophagic markers BECN1 and LC3 (LC3-I and LC3-II), (**b**) the apoptotic marker proCASP3/CASP3 and tubulin protein as loading control, assessed in hMSCs after eighteen days of morphine exposition, in a concentration range from 0 to 1mM. Immediately after the protein transfer, each marker was separated and empty lanes were removed by cutting the blots before the blocking reaction, using 5% bovine serum albumin (BSA) in TBS - Tween buffer (blocking reagent) (Invitrogen, Thermo Fisher Scientific, Monza, Italy) and primary antibody hybridization, carried out with: rabbit anti-human Beclin (Cell Signaling Technologies, Euroclone, Milan, Italy); rabbit anti-human LC3 (Cell Signaling Technologies, Euroclone, Milan, Italy); rabbit anti-human caspase 3 antibody (Cell Signaling Technologies, Euroclone, Milan, Italy); mouse anti-human tubulin antibody (Sigma-Aldrich, St Louis, Missouri, USA).
